# Prediction accuracy of genomic estimated breeding values for fruit traits in cultivated tomato (*Solanum lycopersicum* L.)

**DOI:** 10.1186/s12870-024-04934-8

**Published:** 2024-03-27

**Authors:** Jeyun Yeon, Thuy Tien Phan Nguyen, Minkyung Kim, Sung-Chur Sim

**Affiliations:** 1 Department of Bioindustry and Bioresource Engineering, Sejong University, Seoul, Republic of Korea; 2 Plant Engineering Research Institute, Sejong University, Seoul, Republic of Korea

**Keywords:** Genomic selection, Germplasm collection, Cross-validation, Prediction model, SNP

## Abstract

**Background:**

Genomic selection (GS) is an efficient breeding strategy to improve quantitative traits. It is necessary to calculate genomic estimated breeding values (GEBVs) for GS. This study investigated the prediction accuracy of GEBVs for five fruit traits including fruit weight, fruit width, fruit height, pericarp thickness, and Brix. Two tomato germplasm collections (TGC1 and TGC2) were used as training populations, consisting of 162 and 191 accessions, respectively.

**Results:**

Large phenotypic variations for the fruit traits were found in these collections and the 51K Axiom^™^ SNP array generated confident 31,142 SNPs. Prediction accuracy was evaluated using different cross-validation methods, GS models, and marker sets in three training populations (TGC1, TGC2, and combined). For cross-validation, LOOCV was effective as *k*-fold across traits and training populations. The parametric (RR-BLUP, Bayes A, and Bayesian LASSO) and non-parametric (RKHS, SVM, and random forest) models showed different prediction accuracies (0.594–0.870) between traits and training populations. Of these, random forest was the best model for fruit weight (0.780–0.835), fruit width (0.791–0.865), and pericarp thickness (0.643–0.866). The effect of marker density was trait-dependent and reached a plateau for each trait with 768−12,288 SNPs. Two additional sets of 192 and 96 SNPs from GWAS revealed higher prediction accuracies for the fruit traits compared to the 31,142 SNPs and eight subsets.

**Conclusion:**

Our study explored several factors to increase the prediction accuracy of GEBVs for fruit traits in tomato. The results can facilitate development of advanced GS strategies with cost-effective marker sets for improving fruit traits as well as other traits. Consequently, GS will be successfully applied to accelerate the tomato breeding process for developing elite cultivars.

**Supplementary Information:**

The online version contains supplementary material available at 10.1186/s12870-024-04934-8.

## Background

Tomato (*Solanum lycopersicum* L.) is a major vegetable crop cultivated worldwide and has been intensively studied in the Solanaceae family. Since fruit traits are important in this crop, great efforts have been made for genetic dissection and breeding. Several QTL associated with these traits, including fruit weight, shape, and locule number, have been identified using bi-parental populations and germplasm collections [1–7]. Of these, major QTL have been used to improve fruit traits via marker-assisted selection (MAS) in breeding programs but this approach has a limitation for minor QTL with small effects [8, 9].

Genomic selection (GS) was proposed as an effective breeding strategy for improving complex quantitative traits by predicting genomic estimated breeding values (GEBVs) of individuals [10]. GS provides a way to overcome the limitations of MAS because GEBVs are determined based on effects of genome-wide markers that can capture both major and minor QTL [11–13]. Marker effects are estimated using both genotypic and phenotypic data of a training population in GS models and then are used to predict GEBVs of selection candidates. GS has been successfully implemented in animal breeding programs for increasing genetic gains [14]. With advances in genome sequencing and genotyping technologies, GS has been extensively studied in crop species, especially cereals such as wheat, maize, and rice [15]. For vegetable crops, the prediction accuracy of GEBVs was investigated for fruit traits and capsaicinoid contents in chili pepper [16, 17]. GS was also studied for fruit traits, earliness, heat tolerance, and disease resistance in tomato [18–24]. These studies suggested that GS is a promising tool to accelerate plant breeding cycles for quantitative traits.

Several statistical models for GS have been developed based on parametric and non-parametric methods. These have different assumptions to estimate marker effects for GEBVs and model performance can depend on the genetic architecture of quantitative traits [25, 26]. As parametric models, ridge regression-best linear unbiased prediction (RR-BLUP) and Bayesian models (e.g. BayesA and Bayesian LASSO) have been commonly used for additive genetic effects in crop species [15]. The RR-BLUP model assumes that all markers have common variances with small effects, while the Bayesian models allows different effects and variances of markers with various degrees of shrinkage [10, 27, 28]. The non-parametric models such as reproducing kernel Hilbert space (RKHS), support vector machine (SVM), and random forest (RF) have been known to be better for capturing non-additive genetic effects and multi-variates relative to parametric models [29–31]. For RKHS, the Euclidian genetic distance based Gaussian kernel is used to predict GEBVs with a smoothing parameter to regulate the distribution of marker effects [29, 32]. Based on several kernel methods, SVM can analyze non-linear relationships between phenotypes and genotypes for GS [33]. The RF model uses an ensemble of decision trees and randomly selected subsets of predictor variables as candidates for splitting tree nodes [34, 35]. In addition to the GS model, training population and marker density also affect the prediction accuracy of GEBVs [13, 36–39]. The size and genetic diversity of training populations are important to enable reliable predictions in GS [15, 37, 40–43]. Generally, prediction accuracy increases as training populations are larger. For genetic diversity, high levels of accuracy in GS can be obtained from training populations consisting of individuals with different pedigrees and genetic backgrounds. High-density markers across genome also lead to increase in prediction accuracy by capturing LD between marker and QTL [44, 45]. However, the effect of marker density depends on several factors including species, population types, and traits [46–48].

The present study was conducted to investigate the prediction accuracy of GEBVs for five fruit traits (fruit weight, fruit width, fruit height, pericarp thickness, and Brix) using two tomato germplasm collections (TGC1 and TGC2). These GS panels consisted of 162 and 191 accessions with diverse genetic variations and were independently used as training populations along with a combined population for analysis. Prediction accuracy was evaluated using different cross-validation methods, GS models, and marker sets in three training populations (TGC1, TGC2, and combined). Both parametric and non-parametric models were used to evaluate their performances for the fruit traits. To assess an effective marker density for each trait, eight subsets of markers were generated from the confident 31,142 genome-wide SNPs. In addition, two GWAS-based marker sets of 192 and 96 SNPs were used to improve prediction accuracy with small numbers of markers. The results from our study will accelerate GS in tomato breeding programs by enhancing prediction accuracy with a cost-effective method.

## Results

### Phenotype variation and genetic diversity in training populations

Both TGC1 (*n* = 162) and TGC2 (*n* = 191) showed wide ranges of phenotypic variations for five fruit traits (Table 1). Fruit weight ranged from 17.39 to 186.92 g with a mean of 76.90 g in TGC1 and 12.02 to 262.77 g with a mean of 68.94 g in TGC2. The phenotypic variations of fruit width and fruit height in TGC1 were 24.68–74.36 mm with a mean of 48.83 mm and 29.90–83.19 mm with a mean of 51.48 mm, respectively. These traits showed similar levels of variations in TGC2: 23.17–86.62 mm with a mean of 47.44 mm and 23.11–92.77 mm with a mean of 44.65 mm for fruit height. For pericarp thickness, we observed 2.24–8.60 mm in TGC1 and 3.80–7.22 mm in TGC2 with means of 5.94 mm and 5.47 mm. Brix ranged from 3.84 to 7.86% with a mean of 5.37% in TGC1 and 3.07 to 8.09% with a mean of 5.13% in TGC2. As shown in TGC1 and TGC2, the combined population (*n* = 353) showed substantial phenotypic variations for these fruit traits (Table 1).

**Table 1:** Summary of phenotypic data for five fruit traits in three tomato training populations

Training population	Trait^a,b^	Minimum	Median	Maximum	Mean ± SD^c^
TGC1 (*n* = 162)	FW (g)	17.39	69.87	186.92	76.90 ± 37.06
	FWt (mm)	24.68	47.62	74.36	48.83 ± 11.12
	FH (mm)	29.90	53.10	83.19	51.48 ± 8.94
	PT (mm)	2.24	6.29	8.60	5.94 ± 1.41
	Brix (%)	3.84	5.22	7.86	5.37 ± 0.76
TGC2 (*n* = 191)	FW (g)	12.02	55.30	262.77	68.94 ± 47.31
	FWt (mm)	23.17	46.36	86.62	47.44 ± 13.45
	FH (mm)	23.11	45.17	92.77	44.65 ± 9.58
	PT (mm)	3.80	5.52	7.22	5.47 ± 0.62
	Brix (%)	3.07	5.01	8.09	5.13 ± 0.90
Combined (*n* = 353)	FW (g)	13.70	65.49	262.92	73.31 ± 42.67
	FWt (mm)	23.91	47.17	87.07	48.20 ± 12.42
	FH (mm)	26.44	48.92	95.91	48.06 ± 9.29
	PT (mm)	2.52	5.81	8.28	5.75 ± 1.07
	Brix (%)	3.28	5.13	8.12	5.27 ± 0.83

^a^FW (fruit weight), FWt (fruit width), FH (fruit height), and PT (pericarp thickness)

^b^The phenotypic data of three training populations were corrected for environmental effects using the best linear unbiased prediction (BLUP)

^c^Standard deviation

**Fig. 1:**
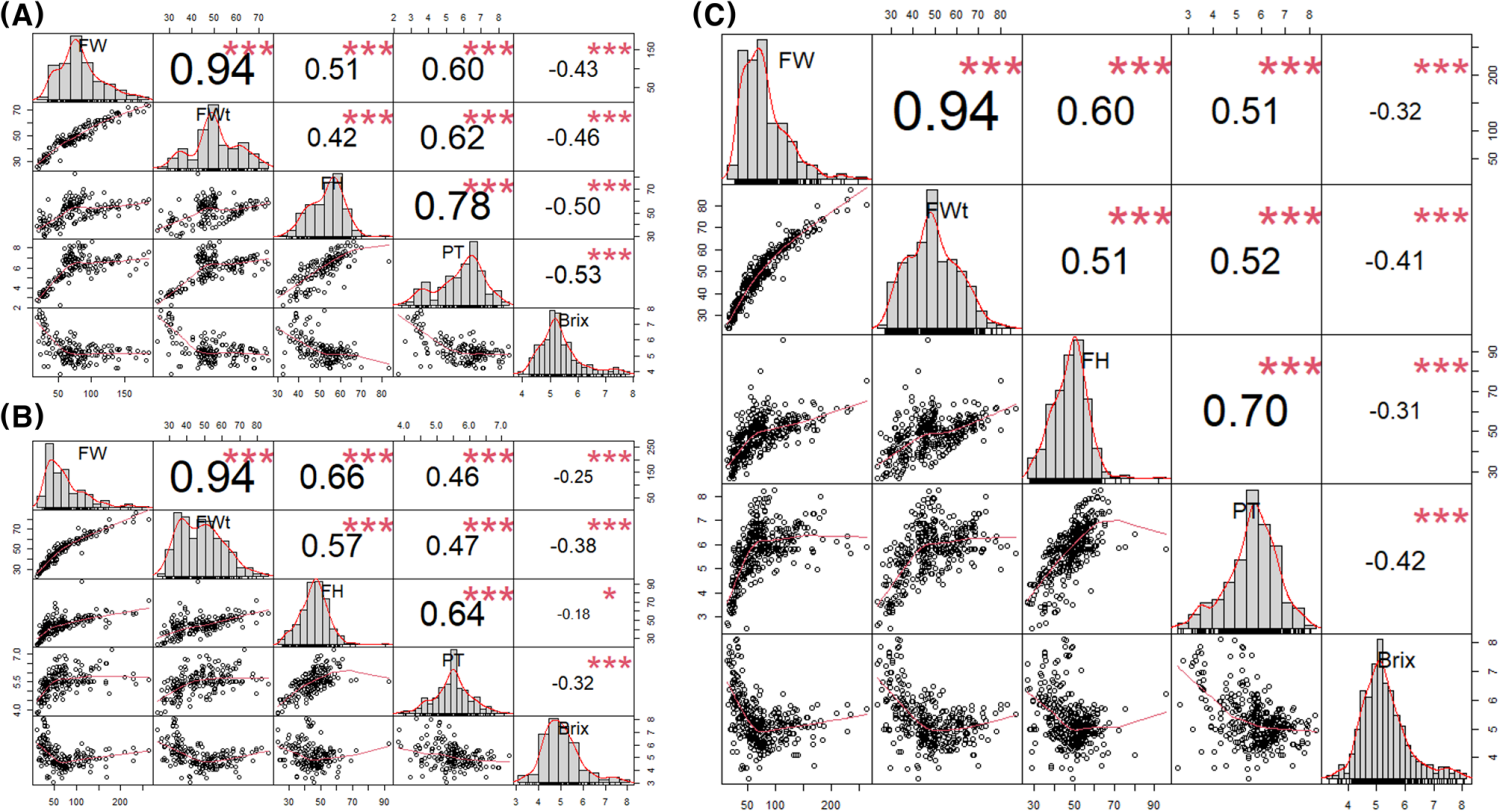
Phenotypic distribution and correlation between five fruit traits in three training populations, (**A**) TGC1 (*n* = 162), (**B**) TGC2 (*n* = 191), and (**C**) combined (*n* = 353). The phenotypic data were corrected for environmental effects using the best linear unbiased prediction (BLUP). Each box shows histograms (diagonal), the Pearson correlation coefficients (upper right diagonal), and pairwise scatter plots (lower left diagonal) between traits. The fruit traits are presented by FW (fruit weight), FWt (fruit width), FH (fruit height), PT (pericarp thickness), and Brix. ***P < 0.001, **P < 0.01, and *P < 0.05

Fruit weight showed different levels of significant correlations at *P* < 0.001 relative to fruit width (0.94), fruit height (0.51–0.66), and pericarp thickness (0.46–0.60) in three training populations (TGC1, TGC2, and combined) (Fig. 1). For fruit height, higher correlation coefficients were found with fruit weight (0.66) and fruit width (0.57) in TGC2 relative to TGC1 (0.51 and 0.42) and the combined population (0.60 and 0.51) at *P* < 0.001. However, pericarp thickness showed the highest levels of correlation with fruit weight, fruit width, and fruit height in TGC1 (Fig. 1). Negative correlation coefficients were found between Brix and the other fruit traits, ranging from − 0.43 (vs. fruit weight) to -0.53 (vs. pericarp thickness) in TGC1 at *P* < 0.001, -0.18 (vs. fruit height) to -0.38 (vs. fruit width) in TGC2 at *P* < 0.05, and − 0.31 (vs. fruit height) to -0.42 (vs. pericarp thickness) in the combined population *P* < 0.001 (Fig. 1).

**Fig. 2:**
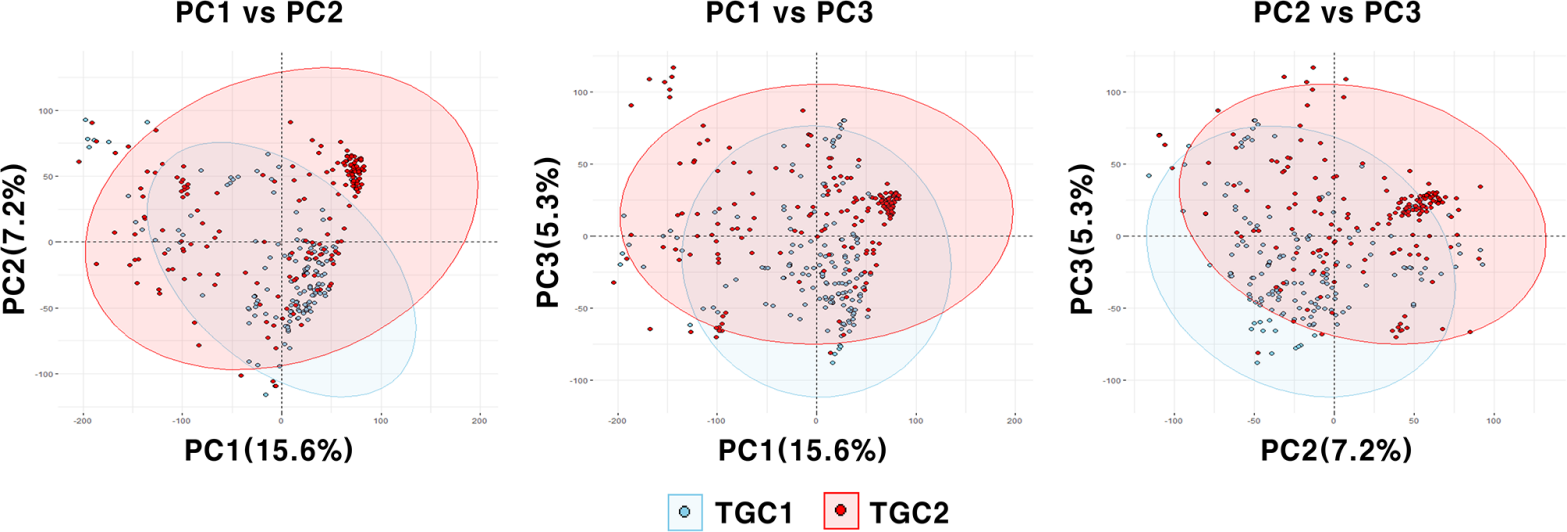
Principal component analysis (PCA) of the 353 accessions from both TGC1 and TGC2 with 95% confidence ellipses. Three principal components (PC1, PC2, and PC3) based on the confident 31,142 SNPs are shown in the plots and the numbers in parenthesis indicate genetic variations explained by each PC

**Fig. 3:**
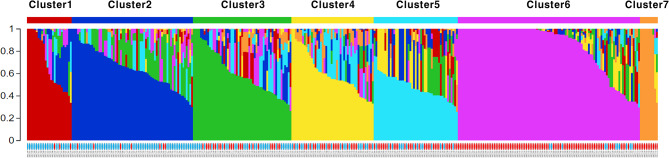
An Inferred population structure in the combined population (*n* = 353) based on the model-based clustering analysis in STRUCTURE v2.3.4. Each accession is shown as a single vertical line, which is partitioned into colored segments in proportion to the estimated membership in each of the seven clusters. Two tomato collections are indicated by blue (TGC1) and red (TGC2) under the bar plot

Genetic diversity in the training populations was evaluated using the confident 31,142 SNPs distributed across 12 chromosomes. Principal component analysis (PCA) indicated diverse genetic backgrounds in both TGC1 and TGC2 (Fig. 2). In addition, genetic differentiation between these training populations was observed based on three PCs, explaining 15.6% of the total variance (PC1), 7.2% (PC2), and 5.3% (PC3). Population structure analysis in the combined population also demonstrated genetic differentiation between TGC1 and TGC2 by separating 353 tomato accessions into seven clusters (Fig. 3). The number of accessions per cluster ranged from 10 (cluster 7) to 102 (cluster 6). Of these, the cluster 2 consisted of 61 TGC1 accessions (89.7%) and seven TGC2 accessions (10.3%). The majority of accessions (88.0%) in the cluster 1 were also derived from TGC1. In contrast, clusters 6 and 7 were represented by TGC2 accessions (89.2% and 100.0%) (Fig. 3 and Table S1). The other clusters showed high levels of mixture with TGC1 and TGC2 accessions (31.9–68.1% for each collection).

### Prediction accuracy of cross-validation methods and genomic selection models

The LOOCV and *k*-fold (*k* = 10 and 5) methods for cross-validation were evaluated in three training populations using the RR-BLUP model. In TGC1, LOOCV showed 0.670 (Brix) to 0.867 (fruit width) of the Pearson correlation coefficients between GEBVs and observed phenotypes (hereafter referred to as prediction accuracy) (Table 2). Similar levels of prediction accuracy were obtained with two *k*-fold methods, ranging from 0.636 (Brix) to 0.859 (fruit width) for 10-fold and 0.652 (Brix) to 0.853 (fruit width) for 5-fold (Table 2). The LOOCV and *k*-fold methods in TGC2 led to higher prediction accuracies for Brix (0.747–0.776) compared to TGC1, but lower prediction accuracies for fruit weight (0.741–0.748), fruit width (0.762–0.766), fruit height (0.687–0.698), and pericarp thickness (0.614–0.618). As shown in TGC1 and TGC2, we found comparable prediction accuracies between the cross-validation methods in the combined population (Table 2). Since LOOCV was effective as *k*-fold for prediction accuracy and has an advantage for computation time, this cross-validation method was used for further analysis in this study.

**Table 2:** Prediction accuracy of cross-validation methods for five fruit traits in three tomato training populations

Training population	Method^a^	Training set size	Prediction accuracy^b^				
			FW^c^	FWt	FH	PT	Brix
TGC1 (*n* = 162)	LOOCV	161	0.822	0.867	0.821	0.856	0.670
	*k*-fold	145 (*k* = 10)	0.823	0.859	0.813	0.847	0.636
		129 (*k* = 5)	0.807	0.853	0.806	0.851	0.652
TGC2 (*n* = 191)	LOOCV	190	0.748	0.766	0.687	0.618	0.776
	*k*-fold	171 (*k* = 10)	0.747	0.765	0.698	0.618	0.747
		152 (*k* = 5)	0.741	0.762	0.687	0.614	0.761
Combined (*n* = 353)	LOOCV	352	0.758	0.802	0.719	0.765	0.736
	*k*-fold	317 (*k* = 10)	0.754	0.798	0.715	0.752	0.723
		282 (*k* = 5)	0.741	0.790	0.703	0.748	0.727

^a^Two cross-validation methods, leave-one-out cross-validation (LOOCV) and *k*-fold (*k* = 10 and 5) were evaluated and each *k* were iterated in 100 different dividing patterns

^b^Prediction accuracy was estimated using the Pearson correlation coefficients between genomic estimated breeding values (GEBVs) and observed phenotypes. The GEBVs were calculated using the confident 31,142 SNPs in the RR-BLUP model

^c^FW (fruit weight), FWt (fruit width), FH (fruit height), and PT (pericarp thickness)

Six GS models, which represent parametric (RR-BLUP, BA, and BL) and non-parametric (RKHS, SVM, and RF) models, were evaluated for prediction accuracy in three training populations. These models showed different accuracies between fruit traits and training populations. In TGC1, RF showed the highest levels of prediction accuracy for fruit weight (0.835), pericarp thickness (0.866), and Brix (0.702), while RKHS was the best model for fruit width (0.870) and fruit height (0.822) (Table 3). Similarly, the highest prediction accuracies were found with RF for fruit weight (0.780), fruit width (0.791), and pericarp thickness (0.643) in TGC2. RKHS and SVM provided better performance for fruit height (0.700) and Brix (0.797) relative to the other models (Table 3). Furthermore, RF revealed the best performance across three traits in the combined population: fruit weight (0.812), fruit width (0.834), and pericarp thickness (0.807) (Table 3 and Table S2). For fruit height and Brix, the highest prediction accuracy was shown with RKHS and SVM, respectively.

**Table 3:** Prediction accuracy of six genomic selection (GS) models for five fruit traits based on the confident 31,142 SNPs in three training populations

GS model^a^		Training population	Prediction accuracy^b^				
			FW^c^	FWt	FH	PT	Brix
Parametric	RR-BLUP	TGC1	0.822	0.867	0.821	0.856	0.670
		TGC2	0.748	0.766	0.687	0.618	0.776
		Combined	0.758	0.802	0.719	0.765	0.736
	BA	TGC1	0.824	0.868	0.821	0.856	0.673
		TGC2	0.744	0.761	0.686	0.624	0.772
		Combined	0.775	0.804	0.715	0.765	0.734
	BL	TGC1	0.816	0.861	0.815	0.853	0.686
		TGC2	0.734	0.779	0.679	0.623	0.779
		Combined	0.766	0.799	0.708	0.748	0.739
Non-parametric	RKHS	TGC1	0.828	0.870	0.822	0.859	0.682
		TGC2	0.758	0.775	0.700	0.625	0.784
		Combined	0.777	0.813	0.738	0.773	0.746
	SVM	TGC1	0.804	0.851	0.797	0.860	0.690
		TGC2	0.755	0.772	0.669	0.594	0.797
		Combined	0.778	0.808	0.723	0.767	0.765
	RF	TGC1	0.835	0.865	0.810	0.866	0.702
		TGC2	0.780	0.791	0.641	0.643	0.778
		Combined	0.812	0.834	0.728	0.807	0.751

^a^Ridge regression-best linear unbiased prediction (RR-BLUP), BayesA (BA), Bayesian LASSO (BL), reproducing kernel Hilbert space (RKHS), support vector machine (SVM), random forest (RF)

^b^Prediction accuracy was estimated using the Pearson correlation coefficients between genomic estimated breeding values (GEBVs) and observed phenotypes

^c^FW (fruit weight), FWt (fruit width), FH (fruit height), and PT (pericarp thickness)

### Genomic selection with different marker sets

**Fig. 4:**
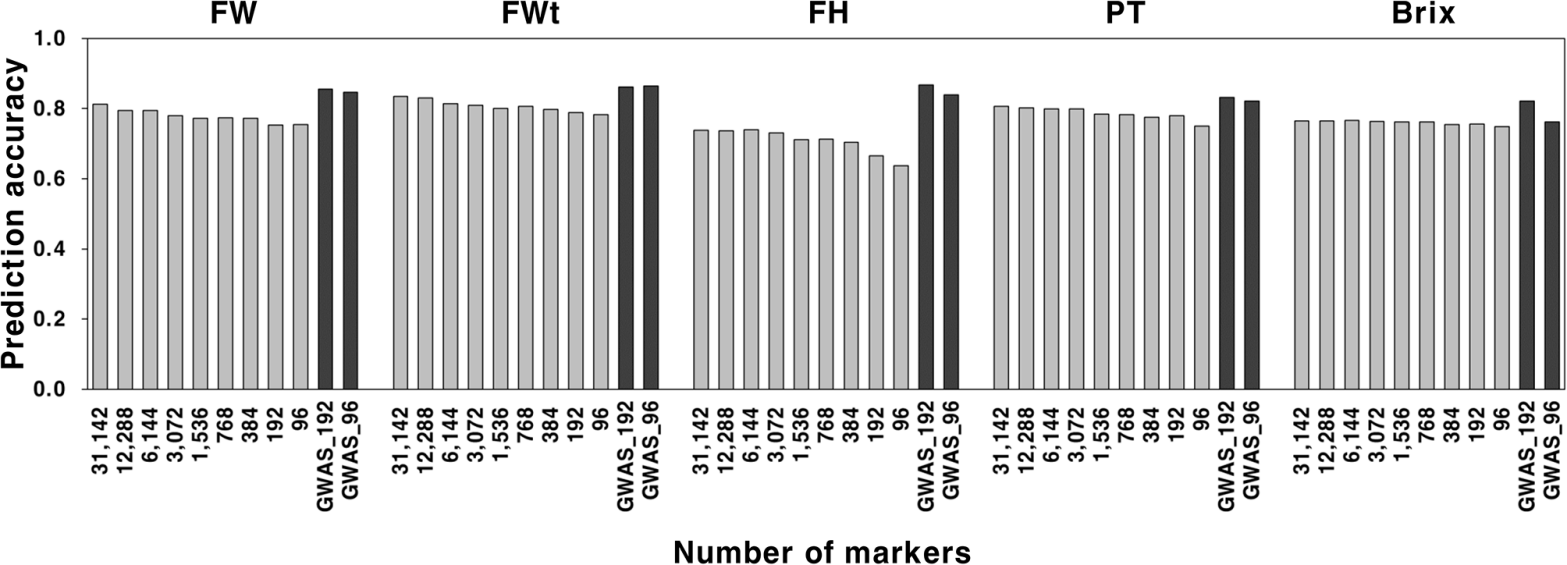
Prediction accuracy of different marker sets for five fruit traits in the combined population (*n* = 353). The eight subsets of SNPs (12,288, 6,144, 3,072, 1,536, 768, 384, 192, and 96) were generated from the confident 31,142 SNPs based on their distributions across 12 chromosomes. Two additional sets (named with GWAS_192 and GWAS_96) were selected based on genome-wide associated study in the combined population. Genomic estimated breeding values (GEBVs) were estimated using the best models: random forest for fruit weight (FW), fruit width (FWt), and pericarp thickness (PT); reproducing kernel Hilbert space (RKHS) for fruit height (FH); and support vector machine for Brix. Prediction accuracy was evaluated based on the Pearson correlation coefficients between GEBVs and observed phenotypes

To assess the effect of marker density for prediction accuracy, the eight subsets of SNPs (12,288, 6,144, 3,072, 1,536, 768, 384, 192, and 96) were generated from the confident 31,142 SNPs. Prediction accuracy was estimated using RF (fruit weight, fruit width, pericarp thickness), RKHS (fruit height), and SVM (Brix) in the combined population. Most of the subsets showed lower accuracies relative to the 31,142 SNPs, ranging from 0.753 to 0.795 (fruit weight), 0.783 to 0.830 (fruit width), 0.637 to 0.740 (fruit height), 0.750 to 0.802 (pericarp thickness), and 0.748 to 0.766 (Brix) (Fig. 4 and Table S3). The number of markers for a plateau ranged from 768 (Brix) to 12,288 (fruit width).

**Fig. 5:**
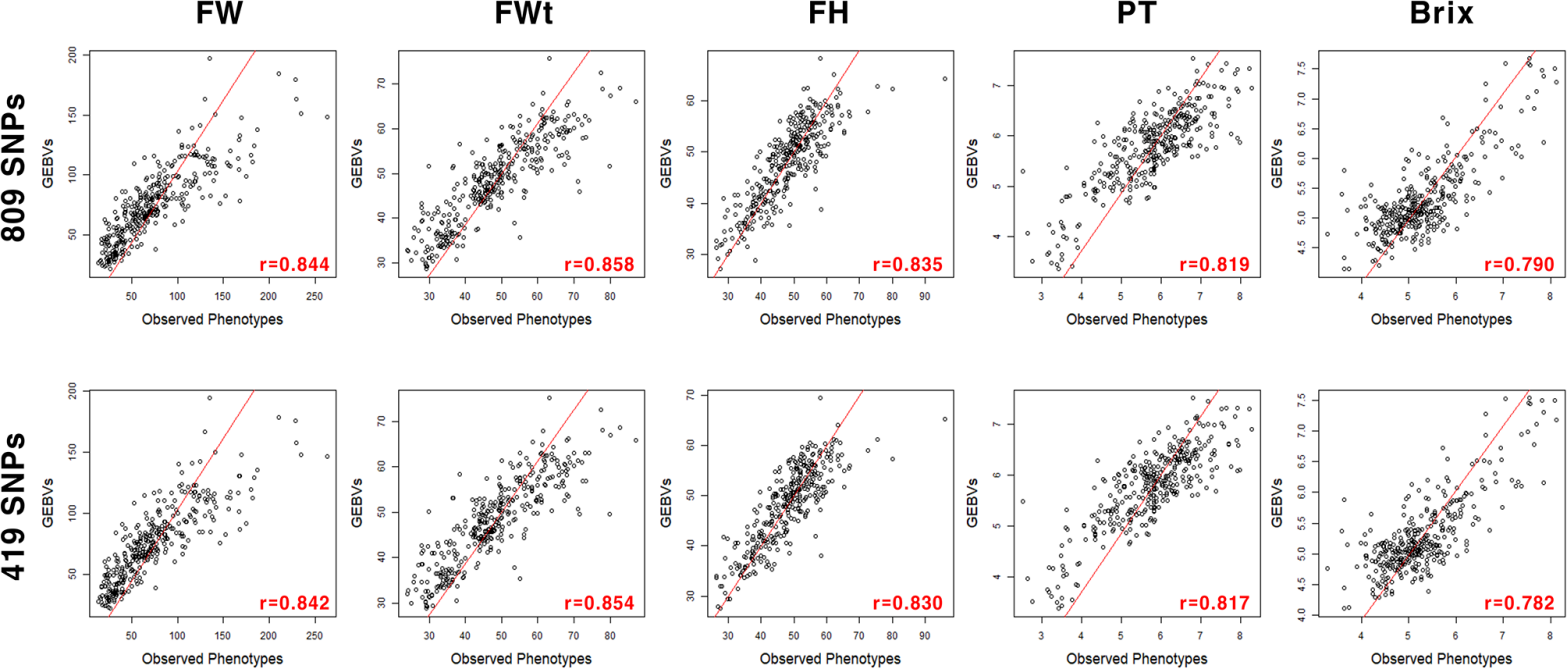
Scatter plots between genomic estimated breeding values (GEBVs) and observed phenotypes for five fruit traits in the combined population (*n* = 353). Two universal marker sets of 809 and 419 SNPs were generated using the 192 and 96 SNPs significantly associated with each trait, respectively. GEBVs were estimated using the best models: random forest for fruit weight (FW), fruit width (FWt), and pericarp thickness (PT); reproducing kernel Hilbert space (RKHS) for fruit height (FH); and support vector machine for Brix

In addition, two SNP sets were developed based on GWAS, which was conducted using the BLUP data of five fruit traits in the combined population. The multi-locus mixed model (MLMM) detected 192 SNPs significantly associated with QTL for each trait at *P* < 0.01 and 96 SNPs at *P* < 0.005 (Fig S1). These GWAS-based subsets showed higher levels of prediction accuracy for the fruit traits relative to the 31,142 SNPs (Fig. 4 and Table S3). The accuracies were 0.821 (Brix) to 0.867 (fruit height) for 192 SNPs and 0.762 (Brix) to 0.865 (fruit width) for 96 SNPs. We also developed two universal sets of 809 and 419 SNPs by combining these SNPs. The first set consisted of 131 common SNPs for two to four traits and 678 trait-specific SNPs including 115 for fruit weight, 109 for fruit width, 142 for fruit height, 162 for pericarp thickness, and 150 for Brix (Table S4). For 419 SNPs, there were 55 common SNPs and 364 trait-specific SNPs including 65 for fruit weight, 57 for fruit width, 79 for fruit height, 86 for pericarp thickness, and 77 for Brix (Table S5). These SNPs were distributed across 12 chromosomes with different numbers of SNPs per chromosome (Fig. S2). The prediction accuracies estimated with RF (fruit weight, fruit width, and pericarp thickness), RKHS (fruit height), and SVM (Brix) ranged from 0.790 (Brix) to 0.858 (fruit width) for 809 SNPs and 0.782 (Brix) to 0.854 (fruit width) for 419 SNPs, which were comparable to those of individual SNP sets (Fig. 5).

## Discussion

Genomic selection (GS) is an emerging breeding method to improve complex quantitative traits using GEBVs in crop species. Successful application of GS depends on accurate GEBVs of breeding lines for target traits. In this study, we investigated the prediction accuracy of GEBVs for five fruit traits using two tomato germplasm collections which consisted of 162 (TGC1) and 191 (TGC2) accessions. These collections were genetically differentiated and showed large phenotypic variations for the fruit traits, respectively, suggesting that these are suitable as training populations for GS analysis. In addition, a large population was generated by combining two collections. Since the phenotypic data of TGC1 and TGC2 were generated in independent field trails, BLUP was used to correct for year and location effects in the combined population. As a result, three training populations were used to assess prediction accuracy for fruit traits with different cross-validation methods, GS models, and market sets in tomato.

For cross-validation, *k*-fold is commonly used to calculate GEBVs by dividing a data set into *k* subsets and then using the *k*-1 subsets for training GS models [49]. Leave-one-out-validation (LOOCV), which is a special case of *k*-fold with *k* = number of observations, has been also used for GS analysis in plants and animals [50–52]. This method is an efficient option for cross-validation with small sample sizes. We found that the prediction accuracies of LOOCV ranged from 0.618 to 0.867, which were comparable to 0.618–0.859 for 10-fold and 0.614–0.853 for 5-fold in the three training populations. In addition, LOOCV required a shorter running time (3.4 h) for 31,142 SNP markers in the RR-BLUP model relative to the 10-fold method with 100 iterations (10 h) using the Intel Core™ i9-9900 K processor (3.60 GHz) and 128 GB RAM. This result suggests that LOOCV is a suitable cross-validation method for developing a GS strategy in tomato breeding programs, depending on population sizes.

Several GS models have been developed to estimate GEBVs with different assumptions [25, 26]. We used six models representing parametric (RR-BLUP, BA, and BL) and non-parametric methods (RKHS, SVM, and RF) with the default parameter settings. Of these, higher prediction accuracies were found in the non-parametric models in three training populations. RF showed higher accuracies (0.807–0.834) for three traits (fruit weight, fruit width, and pericarp thickness) compared to the other models, while RKHS and SVM, were the best models for fruit height and Brix, respectively. As shown in this study, RF provided the highest prediction accuracy for fruit weight in a training population consisting of 96 large-fruited F1 tomato varieties [24]. However, two parametric models (GBLUP and Bayesian LASSO) resulted in better predictions relative to the non-parametric models for soluble solids content. A recent study of pepper (*Capsicum* spp.) found higher accuracies for fruit traits using the non-parametric models such as RKHS and RF relative to the parametric models in a collection of 302 accessions [16]. RKHS was the best model (0.73–0.84) for most of the five traits and RF was also an effective model with high levels of prediction accuracy for fruit width, fruit weight, and pericarp thickness. The non-parametric models have been known to capture non-additive effects such as epistasis and genotype x environment interaction for genomic prediction [15, 37]. For example, RF accounts for both the cumulative effect of individual markers and the effect of all interactions among markers in the model. In this model, decision trees were constructed by categorizing data using multiple predictor variables [34]. For RKHS, a kernel function was used to generate a definite matrix which can be effectively used in a liner model [29, 32]. In contrast, the parametric models are appropriate for traits controlled by additive effects [15, 37]. Moreover, the model performance can also be affected with different parameter settings and thus the effect of optimization would be investigated in future.

Marker density is also an important factor that affects the prediction accuracy of GEBVs for GS. Although genome-wide markers increase prediction accuracy, an effective number of markers for prediction varies with species, population types, and traits [15, 46, 48]. In this study, the eight subsets derived from the 31,142 SNPs were used to investigate the effect of marker density on the fruit traits. We found that prediction accuracies plateaued with different numbers of SNPs (768 − 12,288), depending on traits. This result is consistent with those of previous studies in which no meaningful increases of prediction accuracy were found with large numbers of markers relative to their subsets for fruit traits in tomato and pepper [16, 17, 19, 23]. Our study also demonstrates that the use of markers associated with QTL is a strategy to increase prediction accuracy with small marker sets. The 192 and 96 SNPs, which were derived from GWAS, provided higher accuracies than the 31,142 markers in this study. Increases in prediction accuracy with QTL-based markers were also found in several previous studies. In tomato, higher accuracy for bacterial spot resistance was obtained using only markers significantly associated with QTL compared to the full set of markers as random effects [22]. The 98 SNPs from GWAS increased prediction accuracy for capsaicinoid content relative to 18,029 SNPs in pepper [17]. The effect of QTL-based markers for GS was also found in other crops including maize [53] and soybean [54]. We developed two GWAS-based sets of 809 and 419 SNPs by filtering redundant markers between the trait specific sets of 192 and 96 SNPs. These SNPs resulted in high levels of prediction accuracy across the fruit traits, ranging from 0.790 to 0.858 for 809 SNPs and 0.782 to 0.854 for 419 SNPs, suggesting that these marker sets can be an efficient tool to improve multiple fruit traits simultaneously via GS in tomato breeding programs.

In conclusion, we investigated prediction accuracy of GEBVs for GS using three training populations in tomato. For cross-validation, LOOCV was effective as *k*-fold (*k* = 10 and 5) and showed an advantage for computation time in the training populations with up to 353 accessions. Six GS models showed different prediction accuracies and the highest accuracies were obtained from the non-parametric models, RF (fruit weight, fruit width, and pericarp thickness), RKHS (fruit height), and SVM (Brix) across the training populations. This suggests that the best GS model depends on trait of interest and training population. The effect of marker density was also different between the five fruit traits. Furthermore, two small SNP sets, consisting of 192 and 96 from GWAS, showed higher accuracies compared to the genome-wide 31,142 SNPs. Our results will facilitate GS pipeline development and application in tomato breeding programs.

## Methods

### Plant materials

Two tomato germplasm collections, TGC1 (*n* = 162) and TGC2 (*n* = 191), were used as training populations in this study. The 162 tomato accessions of TGC1 were derived from seven countries including India, China, Turkey, and Israel (Table S1). This collection consisted of determinate and semi-determinate accessions with diverse morphological variations of fruit traits. For TGC2, 98 contemporary breeding lines were assembled from the National Institute of Horticultural and Herbal Science (NIHHS) in Rural Development Administration (RDA), Republic of Korea (ROK). Additional 93 accessions were derived from the National Agrobiodiversity Center (NAC) in RDA, the Germplasm Resources Information Network (GRIN) in the U.S. Department of Agriculture, the C. M. Rick Tomato Genetics Resource Center (TGRC), and Sejong University (Table S1). All of these tomato accessions are indeterminate and also have a broad spectrum of phenotypes for fruit traits, originating from 18 countries including ROK, Russia, USA, Uzbekistan, and China.

### Phenotypic evaluation

Field trials were conducted to evaluate phenotypic variations of fruit weight, fruit width, fruit height, pericarp thickness, and Brix over three years (2018–2020) for TGC1 and two years (2016–2017) for TGC2. Plants were first grown in a greenhouse, and six to seven-week-old seedlings were transplanted into plastic-covered fields (high-tunnel) using a randomized complete block design with three to four replications per genotype. For phenotypic evaluation, fully ripe fruits were harvested from the 2nd -4th flowering clusters and 4–10 fruits per replicate for each genotype were used. Image analysis was conducted using the Tomato Analyzer (TA) v4.0 software [55] for fruit width, fruit height, and pericarp thickness. Fruits were longitudinally and horizontally cut through the center, placed cut-side down on a scanner, and digitalized according to the user manual of TA. For fruit weight, we used average values of fruits per replicate. Brix was measured using a PAL-1 refractometer (ATAGO, WA, USA). The phenotypic data collected from TGC1 and TGC2 were corrected for environmental effects using the best linear unbiased prediction (BLUP) in the R package “lme4” [56], respectively. In addition, these data were combined to generate phenotypic data for a large training population based on BLUP. The resulting phenotypic data for three training populations (TGG1, TGC2, and combined) were separately used for further analysis.

### Genotyping and genetic diversity analysis

Genomic DNA was extracted from fresh and young leaf tissues from four-week-old seedlings using a modified cetyl trimethyl ammonium bromide (CTAB) method [57]. The isolated DNA pellets were resuspended in T1/10E buffer (10 mM Tris-HCl pH 8.0, 0.1 mM EDTA) and their concentrations were adjusted to 50 ng/uL using the NanoDrop™ One spectrophotometer (Thermo Fisher Scientific, Waltham, MA, USA). These DNAs were genotyped using the 51K Axiom^TM^ tomato array with 51,912 SNPs [58] according to the manufacturer’s instructions. For SNP calling, the hybridization signals in the form of CEL files were processed using the Affymetirx® Power Tools software package v1.18. The high-quality SNP were filtered based on < 10% of missing data rate and > 5% of minor allele frequency, and then missing data were imputed using BEAGLE v5 with default parameter setting [59]. The resulting 31,142 SNPs, which were common in TGC1 and TGC2, were used for further analysis.

To evaluate genetic diversity in TGC1 and TGC2, principal component analysis (PCA) was conducted using the prcomp function in R (R core team, 2015) and the results were visualized in the R package “factoextra” [60]. In addition, a population structure in these collections was inferred using the STRUCTURE v2.3.4 program. The model, which allows for admixture and correlated allele frequencies, was used to determine the best K (number of clusters). For this analysis, a series of K (1–10) was tested in 10 independent simulations for each K with a burn-in of 20,000 iterations and a Markov Chain Monte Carlo (MCMC) run length of 100,000 iterations. The best K was then determined using the delta K method [61]. A population structure matrix (Q matrix) was then generated using the membership coefficients of the tomato accessions based on the best K.

### Assessment of prediction accuracy for genomic selection

Two cross-validation methods, leave-one-out cross-validation (LOOCV) and *k*-fold [62], were used to evaluate performance using the ridge regression-best linear unbiased prediction (RR-BLUP) model in three training populations (TGC1, TGC2, and combined). For cross-validation, a training population was divided into training and validation sets, and then GEBVs were calculated for five fruit traits. For *k*-fold, 5 and 10 groups were generated from the training population, respectively. Of these, one group was randomly assigned as a validation set and the other groups were used as a training set. This was iterated in 100 times using the 5 or 10 different dividing patterns to predict GEBVs for each trait. Similarly, a training population with *n* individuals was divided into a training set (*n*-1 individuals) and a validation set (a single dividual) with *n* iterations. Prediction accuracy was determined based on the Pearson correlation coefficients between GEBVs and observed phenotypes for each trait. A cross-validation method was selected based on accuracy and time efficiency for further analysis.

Six genomic selection (GS) models were used to assess the prediction accuracy of GEBVs for five fruit traits in three training populations. Of these, ridge regression-best linear unbiased prediction (RR-BLUP), BayesA (BA), and Bayesian LASSO (BL) were used as parametric models. These models were implemented in the R packages “rrBLUP” version 4.6.2 [63] for RR-BLUP and “BGLR” version 1.1.0 [64] for both BA and BL using the default parameter settings. The burn-in of 500 and run length of 1,500 were used for the Bayesian models. We also included three non-parametric models including reproducing kernel Hilbert space (RKHS), support vector machine (SVM), and random forest (RF). For RKHS, the kinship.BLUP function of rrBLUP was implemented with the Gaussian kernel based on the Euclidean distance between individuals. The radial basis function (RBF) kernel for SVM was used to predict GEBVs with the svm function implemented in the R package “e1071” version 1.7–13 [65]. For this model, the regularization parameter was set to one and both genotypic and phenotypic data were internally scaled to zero mean and unit variance as default. The RF model was implemented in the R package “randomForest” version 4.7–1.1” with decision trees [66]. The default values were used for the number of trees (ntree = 500) and number of SNPs randomly selected at each tree node (mtry = sample size/3), while the minimum node size was set to 10.

To investigate the effect of marker density, eight subsets (12,288, 6,144, 3,072, 1,536, 768, 384, 192, and 96) were generated from the confident 31,142 SNPs, which were filtered from all markers in 51-K SNP array. The SNPs in these subsets were selected based on their distributions across 12 chromosomes. In addition, two marker sets of 192 and 96 SNPs for each fruit trait were derived from genome-wide association studies (GWAS) in the combined population. Marker-trait associations in GWAS were identified using the multi-locus mixed model (MLMM) [67] implemented in genomic association and prediction integrated tool (GAPIT) [68]. To correct population structure and familial relatedness, Q and kinship matrices were used as covariates. The kinship matrix was generated using the VanRaden algorithm [69]. The SNPs associated with QTL were selected based on *P* < 0.01 (192 SNPs) and *P* < 0.005 (96 SNPs). These SNPs were also used to develop a universal set across all of five fruit traits.

### Electronic supplementary material

Below is the link to the electronic supplementary material.

**Supplementary Material 1: Table S1.** Description of 353 tomato accessions used in this study and their membership coefficients (Q matrix) at the best K (K = 7) based on STRUCTURE analysis using 31,142 SNPs

**Supplementary Material 2: Table S2.** Variable importance values of the 31,142 SNP markers in random forest

**Supplementary Material 3: Table S3.** Prediction accuracy of different marker sets for five fruit traits in the combined population (n = 353)

**Supplementary Material 4: Table S4.** The universal set of 809 SNP markers significantly associated with five fruit traits in genome-wide association study

**Supplementary Material 5: Table S5.** The universal set of 419 SNP markers significantly associated with five fruit traits in genome-wide association study

**Supplementary Material 6: Figure S1.** Manhattan plots of genome-wide association study for five fruit traits in the combined population (n=353). A total of 31,142 genome-wide SNPs over 12 chromosomes are shown with gray dots. Horizontal lines in the plots indicate two thresholds (blue for *P* < 0.01 and red for *P* < 0.005) to detect significant marker-trait associations. The red dots represent SNPs that were used to generate GWAS-based sets of 192 and 96 SNPs

**Supplementary Material 7: Figure S2.** Distribution of 809 and 419 GWAS-based SNPs across 12 tomato chromosomes. The red lines indicate the 419 GWAS-based SNPs. The number of SNPs per chromosome are shown at bottom of each chromosome, indicating SNPs for the 419 set in parenthesis

## Data Availability

The data supporting the results in this study are included in this article and its supplementary files, or are available from the corresponding author on reasonable request. The genotypic data of tomato collections are available in the Mendeley Data repository, https://data.mendeley.com/datasets/bxcpc274fh/1.

## Abbreviations

QTL: Quantitative trait loci
MAS: Marker-assisted selection
GS: Genomic selection
GEBVs: Genomic estimated breeding values
RR-BLUP: ridge regression-best linear unbiased prediction
BA: Bayesian A
BL: Bayesian LASSO
SVM: Support vector machine
RKHS: Reproducing kernel Hilbert space
RF: Random forest
TGC: Tomato germplasm collection
SNP: Single nucleotide polymorphism
GWAS: genome-wide association study
LD: linkage disequilibrium
PCA: Principal component analysis
LOOCV: leave-one-out cross-validation
FW: fruit weight
FWt: fruit width
FH: Fruit height
PT: pericarp thickness
BLUP: Best linear unbiased prediction
