# Supplementary figures and images for: Prediction accuracy of genomic estimated breeding values for fruit traits in cultivated tomato (Solanum lycopersicum L.)
Source: BMC Plant Biol. 2024 Mar 27;24:222. doi: 10.1186/s12870-024-04934-8 (PMC10976779; doi:10.1186/s12870-024-04934-8)

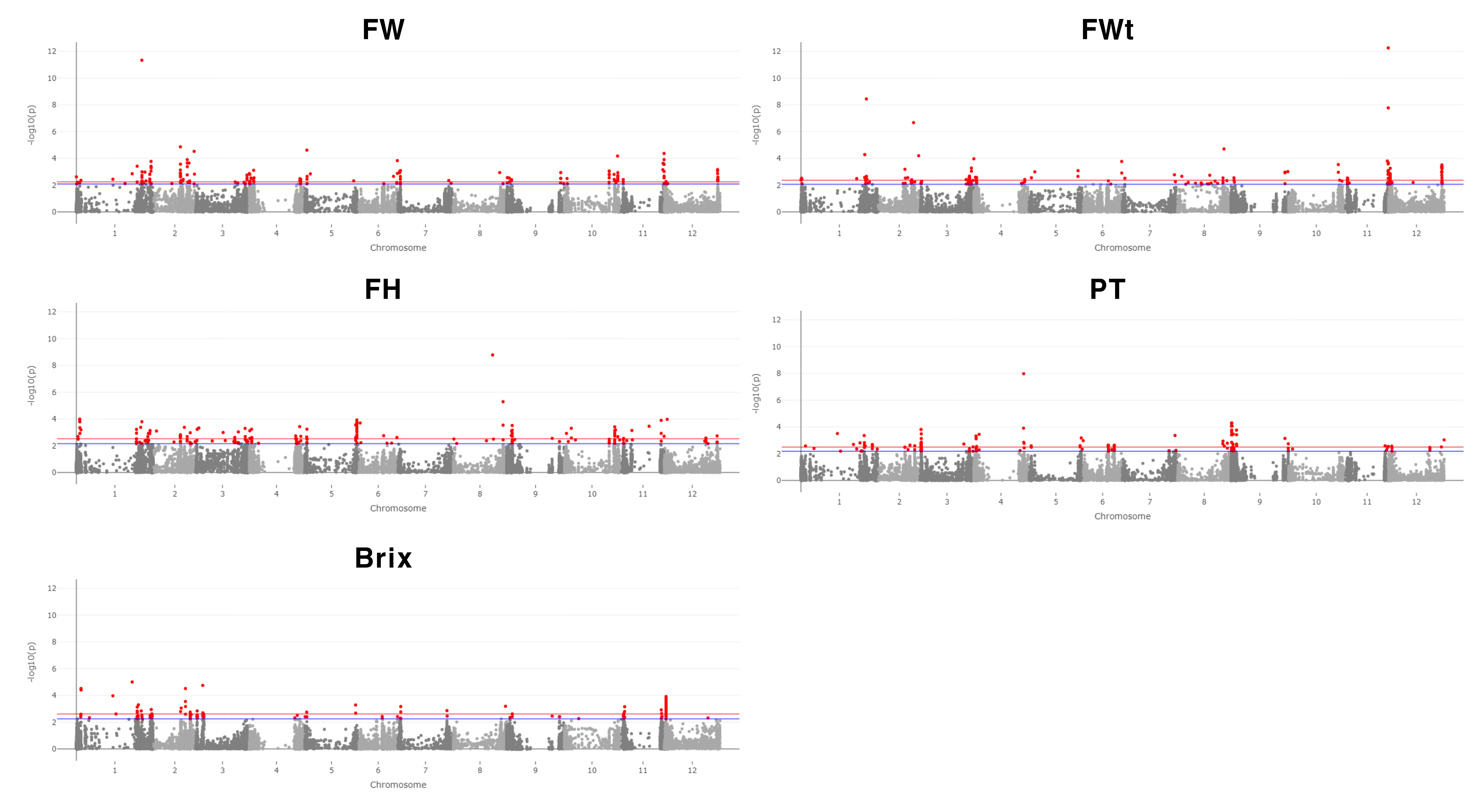

Supplement: Supplementary file 6 — Supplementary Material 6: Figure S1. Manhattan plots of genome-wide association study for five fruit traits in the combined population (n=353). A total of 31,142 genome-wide SNPs over 12 chromosomes are shown with gray dots. Horizontal lines in the plots indicate two thresholds (blue for P < 0.01 and red for P < 0.005) to detect significant marker-trait associations. The red dots represent SNPs that were used to generate GWAS-based sets of 192 and 96 SNPs [file 12870_2024_4934_MOESM6_ESM.tif]

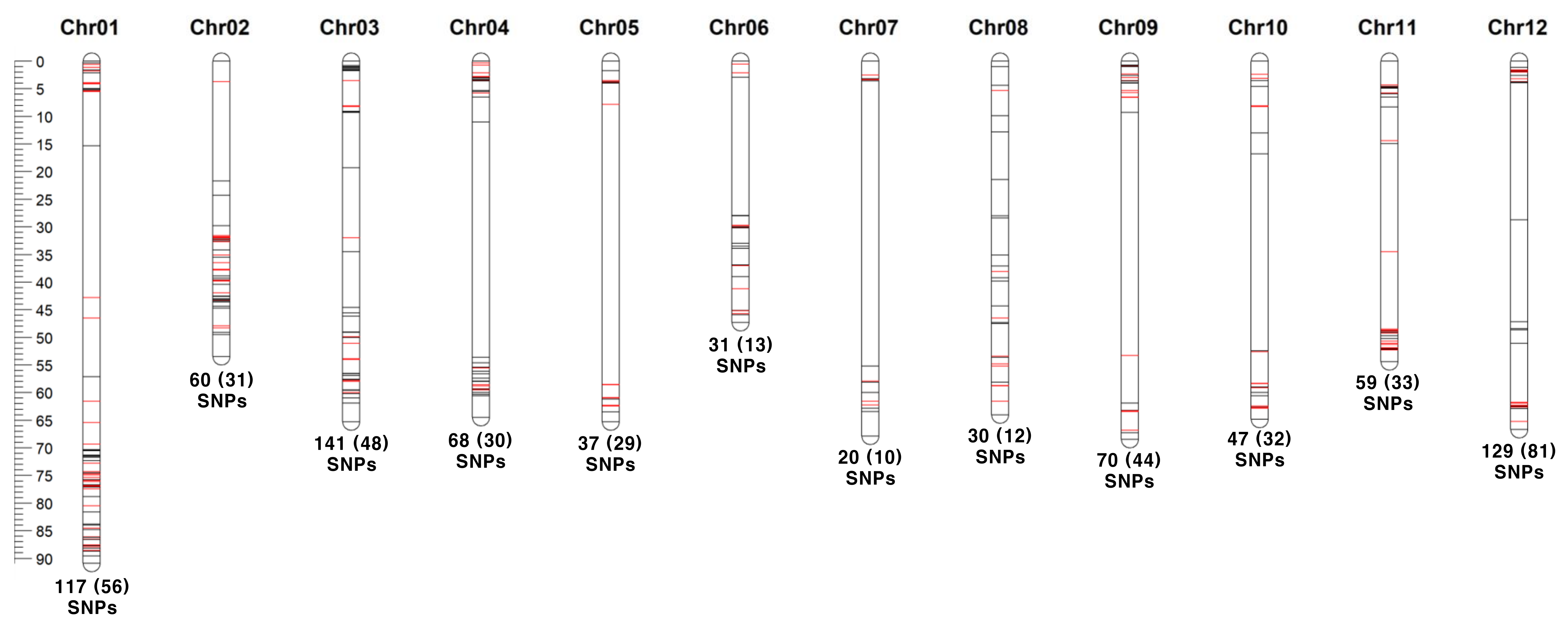

Supplement: Supplementary file 7 — Supplementary Material 7: Figure S2. Distribution of 809 and 419 GWAS-based SNPs across 12 tomato chromosomes. The red lines indicate the 419 GWAS-based SNPs. The number of SNPs per chromosome are shown at bottom of each chromosome, indicating SNPs for the 419 set in parenthesis [file 12870_2024_4934_MOESM7_ESM.tif]
